# Latest advances in the study of non-coding RNA-mediated circadian rhythm disorders causing endometrial cancer

**DOI:** 10.3389/fonc.2023.1277543

**Published:** 2023-11-22

**Authors:** Ling-tao Zheng, Shao-rong Chen, Liang-yu Zhou, Qiao-yi Huang, Jia-ming Chen, Wei-hong Chen, Shu Lin, Qi-yang Shi

**Affiliations:** ^1^ Department of Gynaecology and Obstetrics, The Second Affiliated Hospital of Fujian Medical University, Quanzhou, Fujian, China; ^2^ Centre of Neurological and Metabolic Research, The Second Affiliated Hospital of Fujian Medical University, Quanzhou, Fujian, China; ^3^ Group of Neuroendocrinology, Garvan Institute of Medical Research, Sydney, NSW, Australia

**Keywords:** endometrial cancer, circadian rhythm disorders, non-coding RNAs, clock genes, miRNA, lncRNA

## Abstract

Endometrial cancer (EC) is one of the most common gynecological cancers, and its risk factors include obesity and metabolic, genetic, and other factors. Recently, the circadian rhythm has also been shown to be associated with EC, as the severity of EC was found to be related to night work and rhythm disorders. Therefore, circadian rhythm disorders (CRDs) may be one of the metabolic diseases underlying EC. Changes in the circadian rhythm are regulated by clock genes (CGs), which in turn are regulated by non-coding RNAs (ncRNAs). More importantly, the mechanism of EC caused by ncRNA-mediated CRDs is gradually being unraveled. Here, we review existing studies and reports and explore the relationship between EC, CRDs, and ncRNAs.

## 1 Introduction

Endometrial cancer (EC) is a malignancy of the endometrial epithelium. The annual incidence of EC is very high. Worldwide, 417,367 cases of EC were reported in 2020, making it the sixth most common cancer among women (1). Continuous exposure to exogenous or endogenous estrogen without progesterone antagonism is a risk factor for endometrial cancer. Other risk factors, such as obesity, tamoxifen use, insulin resistance, type 2 diabetes, and polycystic ovary syndrome, can increase the risk of EC (2). The main symptom of EC is abnormal uterine bleeding, which can be accompanied by vaginal secretions and uterine infection (3). When a patient presents with any of these symptoms, abdominal and pelvic examinations should be considered (4). The primary clinical treatment for EC is surgery, including total hysterectomy, bilateral salpingo-oophorectomy, and adjuvant therapy. However, the 5-year survival rates of patients with stage IVA and IVB EC are only 17% and 15%, respectively, although 67% of these patients display early signs of the disease (5). Therefore, finding a new treatment strategy is urgent.

The circadian rhythm is a stable regulatory system in the human body and is regulated by several hormones, particularly melatonin (MLT). Some studies have shown that MLT is involved in the regulation of epithelial-mesenchymal transformation and subsequent tumor invasion (6–10) as well as in inhibiting osteosarcoma (6) and ovarian cancer stem cells (7). At the molecular level, circadian rhythms are regulated by clock genes (CGs). Many diseases are caused by the abnormal expression of these genes, including cancer, endocrine, cardiovascular, and psychological diseases (11, 12). As a genetic disease, cancer is caused by uncontrolled growth and one reason for this is changes in circadian pathway genes (13). Furthermore, non-coding RNAs (ncRNAs), including microRNAs (miRNAs), long non-coding RNAs (lncRNAs), and circular RNAs (circRNAs), are involved in the regulation of CGs, such as miR-576-5p (14), miR-126-5p (15).

In this paper, we reviewed the latest progress in EC caused by CRDs and mediated by ncRNAs. Additionally, we attempted to summarize the relationship between ncRNAs, circadian rhythms, and EC.

## 2 Endometrial cancer

Historically, proliferative lesions that occur in glands without cytological atypia are called “hyperplasia” and have a 2% cancer risk, while those with cytological atypia are called “atypical hyperplasia” and have a 23% cancer risk (16). Endometrial intraepithelial neoplasia (EIN) is now recognized to precede atypical endometrial hyperplasia and is considered a precursor lesion of endometrial cancer (17).

The etiology of EC is not completely clear; however, it includes a variety of risk factors, such as BMI, as analyzed by Aune et al. (18). Using data from 22,320 cases, high BMI at 18–25 years of age, waist and hip circumferences, waist-to-hip ratio, height, and weight gain (over 10kg) were associated with an increased risk of EC. In other words, there is a positive correlation between body fat, weight gain, height, and the risk of EC. Renehan et al. (19) also reported that every 5 kg/m^2^ increase in BMI raises the risk of developing EC. Fisher et al. (20) reported that although tamoxifen reduced the incidence of breast cancer, it increased the risk of EC. Crosbie et al. (2) reported that insulin resistance, hyperinsulinemia, type 2 diabetes, and polycystic ovary syndrome (PCOS) could promote endometrial hyperplasia, which might be associated with EC.

Estrogen promotes endometrial hyperplasia; periodic menstruation and estrogen-antagonistic progesterone work together to maintain endometrial health. In obese women, the adipose tissue converts adrenal androgen to estrogen, forming a hyperestrogenic state (2). This state may interfere with the normal proliferation of the endometrium and increase the risk of EC. Additionally, Modugno et al. (21) showed that obesity is a chronic pro-inflammatory state that promotes the development of an inflammatory microenvironment and is accompanied by high levels of circulating c-reactive protein (CRP), interleukin-6 (IL-6), and tumor necrosis factor-alpha (TNF-α). These inflammatory markers may mediate the changes in the endometrial immune microenvironment.

**Figure 1:**
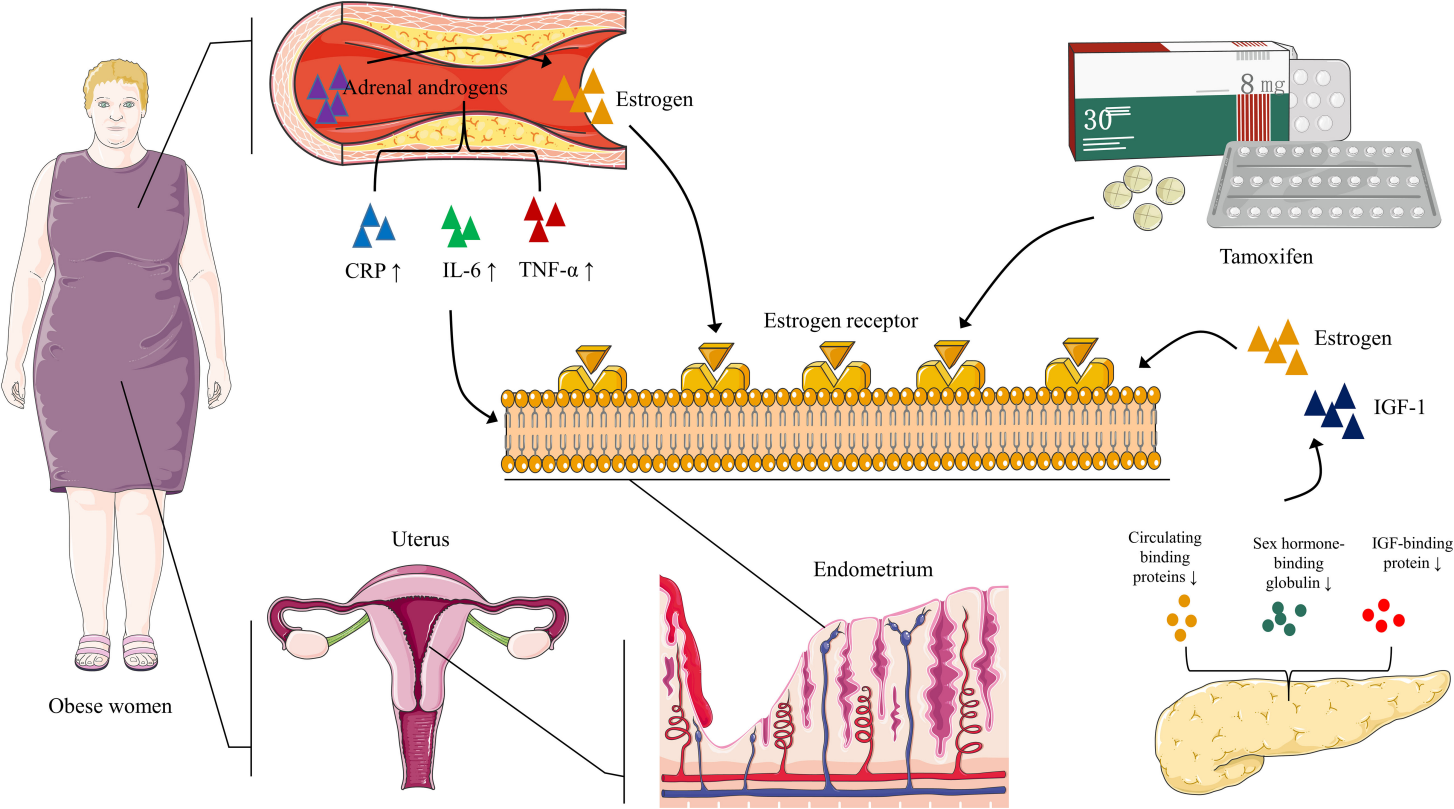
EC: pathways to carcinogenesis In obese women, adipose tissue aromatizes adrenal androgens into estrogen, leading to a hyperestrogenic state. Obesity also promotes high circulating CRP, IL-6, and TNF-α levels, which may be associated with changes in the endometrial immune microenvironment. Prolonged tamoxifen use promotes estrogen binding to its receptor, further enhancing the hyperestrogenic state. Insulin resistance, hyperinsulinemia, type 2 diabetes, and PCOS inhibit circulating binding proteins of estrogen and insulin-like growth factor (IGF)-1, sex hormone-binding globulin, and IGF-binding protein. As a result, estrogen and IGF-1 bioavailability increases, stimulating the endometrium.

Tamoxifen can stimulate endometrial hyperplasia. The stimulating effect of the long-term use of tamoxifen may be the mechanism that increases the risk of EC (20). Similarly, insulin resistance, hyperinsulinemia, type 2 diabetes, and PCOS can reduce the circulation levels of estrogen-binding proteins, insulin-like growth factor (IGF)-1, sex hormone-binding globulin, and IGF-binding protein, and improve their efficiency to stimulate endometrial growth (2) (**Figure 1**).

Total hysterectomy and bilateral salpingo-oophorectomy are cornerstones of EC treatment and can be performed using open or minimally invasive techniques. Minimally invasive surgery is the first treatment choice for early-stage EC when the uterus is completely resectable. Minimally invasive surgery has the advantages of a short hospital stay, less blood loss, less pain, and low perioperative incidence (22–25).

## 3 Circadian rhythm disorders

Sleep and wakefulness have distinct circadian rhythms and are two bodily functional states. Sleep restores vitality, strengthens the immune system, maintains brain and heart health, consolidates memory (26). The circadian rhythm regulates the sleep cycle, and an appropriate circadian rhythm results in normal cell growth and survival. Various factors regulate circadian rhythms, one of the most important of which is MLT. Light-generated neural signals regulate MLT metabolism via the retinohypothalamic tract (RHT)-suprachiasmatic nucleus (SCN)-paraventricular nucleus (PVN)-brainstem-spinal cord (levels T1–T3)-superior cervical ganglion (SCG)-pineal gland pathway (27). Since MLT promotes sleep and reduces daily activity performance, inappropriate light stimulation can produce circadian rhythm disorders (CRDs) and disrupt MLT metabolism in the pineal gland (27).

Sun et al. demonstrated an increase in the number of macrophages in tissues and organs during circadian rhythm disturbances. Several studies have suggested a potential link between circadian rhythms and the cell division cycle (28–30) as well as malignancy (31). This occurs when the timing of daily activities is out of sync with an individual’s innate chronotype (32). CRDs may cause defects in the regulation of cell proliferation (33). For example, disturbances in circadian rhythms caused by night work may increase the risk of breast and prostate cancer (34–36) as well as EC (32, 37).

### 3.1 Clock genes

The first clock gene discovered in *Drosophila* is called the “period” gene (38, 39). There are at least 12 known CGs in mammals: Period1 (*PER1*), Period2 (*PER2*), Period3 (*PER3*), Cryptochrome 1 (*CRY1*), Cryptochrome 2 (*CRY2*), Circadian Locomotor Output Cycles Kaput (*CLOCK*), the transcription factor Aryl Hydrocarbon Receptor Nuclear Translocator-Like (*ARNTL*), Timeless (*TIM*) (13), Retinoic acid-related Orphan Nuclear Receptor (*ROR*) (40–42), Neuronal PAS domain protein-2 (*NPAS2*) (42, 43), Nuclear Receptor Subfamily 1 Group D members 1 and 2 (*NR1D1* and *NR1D2*, respectively, also known as REV-ERB alpha and beta, respectively) (42, 44), and Casein Kinase I Epsilon (*CSNK1E*) (42, 45). Among these, *PER1*, *PER2*, and *NPAS2* are associated with EC (46, 47). AT-rich interaction domain 1A (*ARID1A*) may be involved in the progression of EC by regulating the CGs *BHLHE41* and *ARNTL* (48). The following sections discuss the relationship between CGs and EC.

#### 3.1.1 Period1 and period2

*PER1* and *PER2* may be involved in mechanisms underlying EC onset and progression. Wang et al. (46) found that a high expression of *PER1* and *PER2* was associated with a better prognosis in EC. In their study, western blotting showed that the expression of PER1 and PER2 decreased in the rhythm group, whereas the expression of breast carcinoma amplified sequence 4 (BCAS4), tubulin beta-2B chain (TUBB2B), and Roof Plate-Specific Spondin-4 (RSPO4) increased in the breast cancer group. The high expression of PER1 indicates that the survival time of patients with EC is longer, while the high expression of TUBB2B indicates a lower survival rate. TUBB2B is related to diffuse and symmetrical aberrations in cerebral cortex development, and its importance in the central nervous system reveals its potential role in regulating circadian rhythms. In addition, they transfected Ishikawa cells with overexpressed PER1 plasmid and found that the apoptosis rate was significantly increased after 24h, and cell invasion was disturbed after 24h and 48h (46). A loss of *PER* expression suppresses the diurnal oscillation of decidualized human endometrial stromal cells (49). It is reported that *BCAS4*, *TUBB2B*, and *RSPO4* regulate cancer development by interacting with other proteins (50–52). All in all, the severity of EC is associated with CRDs, and factors such as the CGs PER1 and PER2 may regulate the mechanisms of EC onset and development.

#### 3.1.2 AT-rich interaction domain 1A

The switch/sucrose non-fermentable (SWI/SNF) complex is a nucleosome-remodeling factor found in both eukaryotes and prokaryotes (53). Through transcriptional control, it participates in the regulation of gene expression and is essential for cancer growth (54). SWI/SNF is a multi-subunit complex that includes AT-rich interaction domain 1A (*ARID1A*). *ARID1A* is one of the most commonly mutated genes in human cancers, such as colorectal cancer (55, 56), gastric cancer (57, 58), pancreatic cancer (59), esophageal adenocarcinoma (60), liver cancer (61), ovarian clear cell carcinoma (62), and endometrioid carcinoma (63–65). As reported by Hanyang Hu et al. (48), *ARID1A* regulates the binding of ER to clock gene enhancers in EC. *ARID1A* depletion affects chromatin accessibility and ER binding in enhancers, leading for the downregulation of CGs *ARNTL* and *BHLHE41*, eventually favoring attenuation of endometrial cancer cell proliferation and metastasis. In addition, a decreased *ARID1A* expression was linked to shorter progression-free survival in patients with endometrial-associated cancer. In summary, *ARID1A* and circadian rhythm genes can be regarded as novel diagnostic markers and potential targets for the treatment of EC (53).

#### 3.1.3 Neuronal PAS domain protein-2

*NPAS2*, the longest CG in humans, is a mammalian transcription factor with a length of 176.68 kb (47). The PAS domain of *NPAS2* binds to heme as a prosthetic group, making heme-based signal transduction possible, thereby playing a key role in generating circadian rhythms (66). *NPAS2* participates in the cell cycle and the DNA damage response (67). A recent study showed that high *NPAS2* expression is associated with poor survival in patients with EC (47). The data from that study show that *NPAS2* is positively correlated with poor prognosis in EC. In addition, overexpression of *NPAS2* significantly induces the proliferation of Ishikawa cells, while silenced *NPAS2* inhibits the growth of AN3CA cells, and these situations are likely due to the influence of *NPAS2* expression on the G1 and S phases of the cell cycle. This suggests that *NPAS2* can be used as an indicator for the diagnosis and treatment of EC. Moreover, the researchers analyzed and predicted the expression correlation between miRNAs and *NPAS2* in UCEC using the starBase database and found that *NPAS2* was negatively correlated with *miR-17-5p* (R=-0.119, p=2.09E-02) and *miR-93-5p* (R=-0.091, p=7.96E-02), and positively correlated with *miR-106a-5p* (R=0.111, p=3.21E-02) and *miR-381-3p* (R=0.198, p=1.11E-04) (47).

To sum up, *PER1* and *PER2* may regulate EC pathogenesis and progression, *ARID1A* affects EC cell growth and metastasis, and *NPAS2* affects EC cell proliferation and apoptosis. Focusing on these CGs and exploring corresponding targeted therapy may lead to a potential tool for improving the effectiveness of EC therapy (**Table 1**).

**Table 1:** CGs, their expression in EC, and the corresponding prognosis.

Clock genes	Expression level	Prognosis	References
*PER1 and PER2*	Low	Poor	(46)
*ARID1A*	High		(48)
*NPAS2*	High		(47)

In EC cells, CGs *PER1*, and *PER2* were at low expression levels, while *ARID1A* and *NPAS2* were at high expression levels. The expression levels of all these genes indicate the poor prognosis of EC.

## 4 Endometrial cancer and circadian rhythm disorders

A study showed that age, education, smoking, type of work, marital status, fertility, menopause, gynecological history, hypertension, and shift time were all related to the severity of EC (46). When uncontrollable factors (such as age and menopause) were excluded, the correlation between rhythm-related factors and EC was the strongest (R ≈ 0.1). In order to control the diurnal functioning of the whole body, the circadian rhythm makes the behavior pattern consistent with ambient light and darkness, supporting body function by predicting and coordinating the necessary metabolic procedures (68). Disorders of the circadian rhythm disrupt the metabolic balance in the body. Endometrial proliferation, secretion, and shedding occur periodically, and the disruption of this cycle elevates the risk of disease. Working at night is one such case. Viswanathan et al. (69) reported that night shift work might increase the risk of EC. Besides, Von Behren et al. (32) explored the relationship between EC and chronotypes and found that post-menopausal women with evening chronotypes were more likely to develop EC, especially those with a body mass index (BMI) of 30 or higher. According to a study of sleep/night shift characteristics of patients with EC conducted by Wang et al. (46), the severity of EC is associated with night shift and rhythm disorders. In addition, people who work at night are exposed to inappropriately timed light, causing cortisol, body temperature, and MLT rhythms to be out of sync (70). As discussed above, the effect of CRDs on EC is ultimately mediated by CGs.

## 5 Non-coding RNAs

NcRNAs affect circadian rhythms through the gene-effector protein-circadian rhythm axis. NcRNAs are mainly composed of miRNAs, lncRNAs, and circRNAs (71, 72), and they play an important role in tumor development (73, 74). Here, we discuss the influence of miRNAs and lncRNAs on CRDs. MiRNAs are small ncRNAs, 19–24 nucleotides long, whereas lncRNAs are longer than 200 nucleotides. LncRNAs control gene expression by altering the function of transcription, splicing, translation, or miRNAs (75). Ray et al. (13) found that a subset of ncRNAs changes in cancer tissue, with target sites on certain CG mRNAs that can directly influence the abundance of these clock genes; another subset of ncRNAs targets specific oncogenes or tumor suppressor genes and is directly regulated by CGs. The potential use of ncRNAs in disease diagnosis has become widespread. Gharib et al. (76) examined the levels of *miR-31* in 100 patients with breast cancer and their adjacent normal breast tissues using RT-PCR and concluded that *miR-31* is expressed at low levels in breast cancer. Similarly, Zhao et al. (77) concluded that serum *miR-205-5p* is a valuable biomarker for lung cancer diagnosis because it promotes the proliferation and metastasis of lung cancer cells by regulating TP53INP1. Herein, we summarize the information that has become available in recent years.

By selectively targeting the ZBT4/Sp1 axis, *miR-576-5p* may affect the ability of EC cells to proliferate, migrate, and invade (14). C*irc_0002577* downregulates *miR-126-5p* in concert with MACC1 to promote EC invasion and metastasis (15). *MiR-1271-5p* overexpression prevents EC cell proliferation, migration, and invasion by targeting its downstream target, CTNND1, and induces cell death (78). *MiR-202-5p* (79), *miR-197* (80), *miR-298* (81), and *miR-105* (82) have similar effects to *miR-1271-5p* and can target downstream genes to prevent the proliferation, migration, and invasion of EC cells. CLOCK-controlled *miR-455-5p* regulates circadian rhythms by accelerating the degradation of clock mRNA (83). In short, the above-mentioned miRNAs related to CGs are usually stable in normal tissues but are irregularly expressed in abnormal tissues, especially in tumors (84). Nonetheless, we did not observe many EC-related miRNAs in the data we collected. Future studies should explore additional miRNAs related to EC and CRDs.

**Figure 2:**
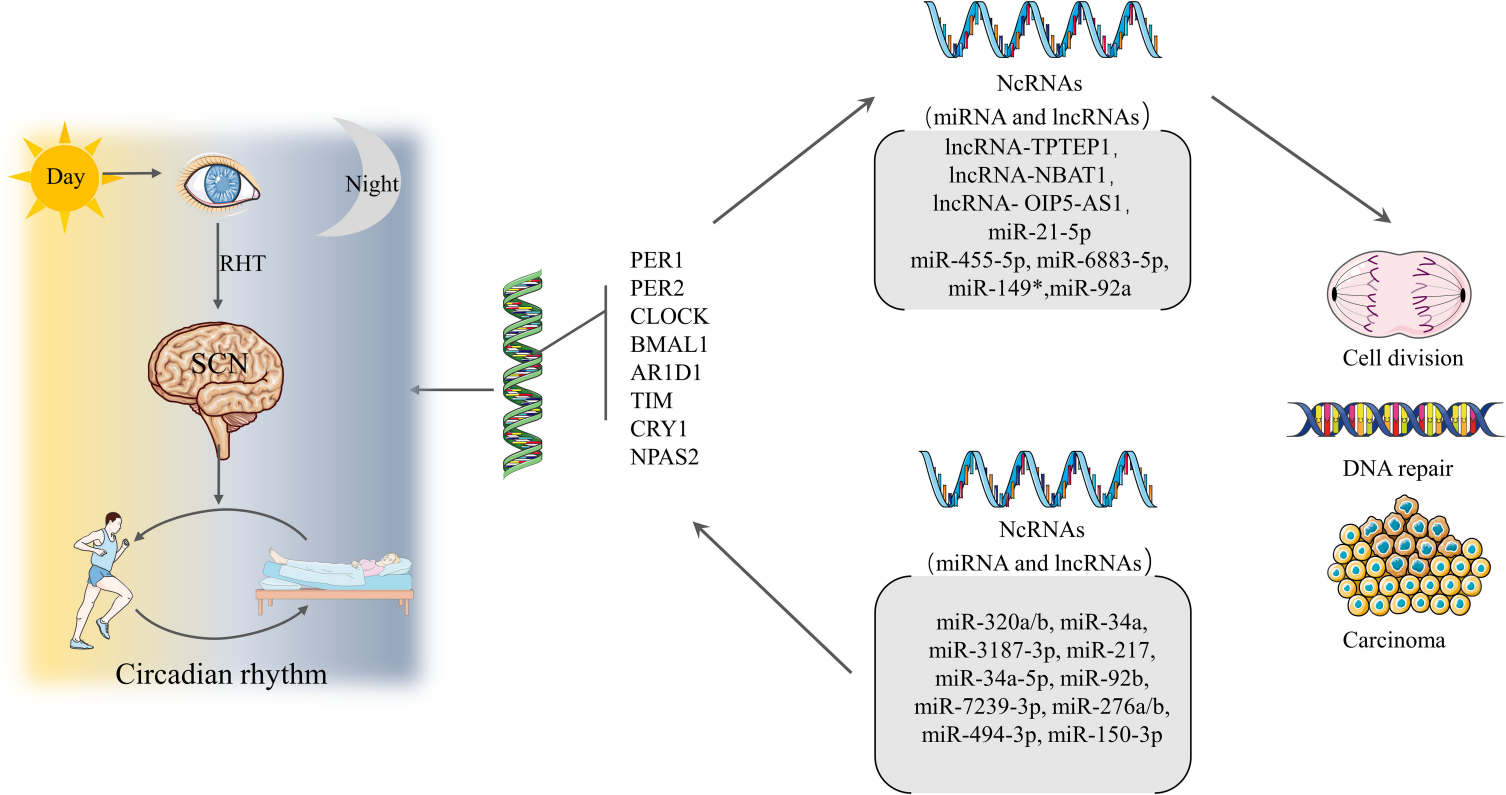
Formation of circadian rhythms and associated regulation Light travels through the retina and through the RHT to the SCN, forming the circadian rhythm that guides the mode of operation of daytime activity and nighttime rest. Circadian rhythms are regulated by CGs. A properly functioning CGs can direct ncRNAs to regulate cell division and DNA repair, while dysregulation can lead to cancer. Conversely, ncRNAs can affect the expression of CGs leading to CRDs and a range of diseases.

LncRNAs also play a important role in EC, as lncRNA binding to miRNAs promotes or inhibits the proliferation of EC cells. For example, the lncRNA *OIP5-AS1* inhibits the proliferation and invasion of EC cells by suppressing *miR-200C-3p*, which in turn regulates PTEN/AKT (85). Thus, lncRNAs, like miRNAs, play an indispensable role in the development of EC. Again, from the information collected, we did not observe many lncRNAs associated with EC. Further exploration of the interaction of lncRNAs and miRNAs with EC will help discover new therapeutic options for treating EC. To summarize the information we collected, we have compiled **Figure 2**.

## 6 Discussion

**Figure 3:**
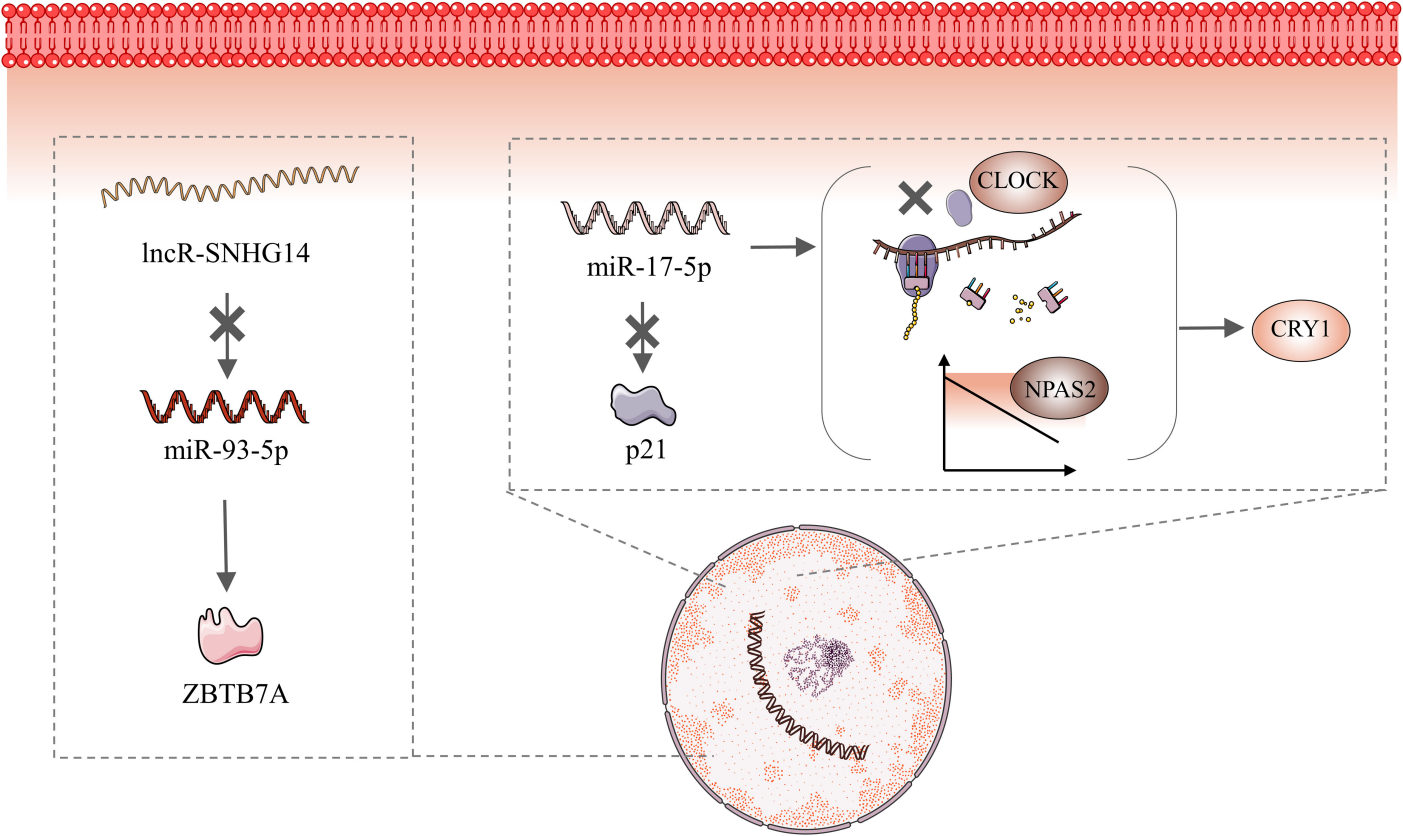
Molecular Mechanisms of *MiR-17-5P* and *miR-93-5 PMiR-17-5P* maintains circadian stability via inhibiting *CLOCK* translation, downregulating *NAPS2* levels, and increasing *CRY1* expression. In addition, *miR-17-5p* directly targets p21 to affect the migration and invasion of endometrial cancer cells. Elevated SNHG14 expression inhibits miR-93-5P, which then directly targets ZBTB7A.

The biological clock is a 24-h self-service oscillator controlled by CGs. Every cell in the human body has a day-night oscillator controlled by a master clock. This oscillator provides rhythmicity to specific cells and organs through rate-limiting metabolic program stages (68). The breakdown of the circadian rhythm disrupts the rhythmic nature of every cell and organ, resulting in a wide range of diseases. The circadian rhythm is regulated by CGs. The disorder of circadian rhythm is usually accompanied by abnormal expression of CGs, which also involves ncRNAs. This review summarizes recent perspectives on EC and CRDs, and collects relevant CGs and ncRNAs, including *PER1*, *PER2*, *NAPS2*, and *ARID1A*. However, based on our review, the only ncRNAs associated with CGs and EC are *miR-17-5p, miR-93-5p,* and *lncRNA SNHG14. MiR-17-5p* expression is negatively correlated with *NAPS2* expression (47). Gao et al. (86) showed that miR-17-5p inhibited *CLOCK* translation, downregulated *NAPS2* levels, and increased *CRY1* expression. Furthermore, *miR-17-5p* directly targeted p21 to affect the migration and invasion of EC cells (87). Zhang et al. (88) showed that the lncRNA *SNHG14* inhibited EC migration and invasion via the *miR-93-5p*/ZBTB7A axis. In other words, *SNHG14* expression negatively correlated with *miR-93-5p*, and high *SNHG14* expression inhibited EC development (**Figure 3**).

From existing references, it can be seen that ncRNAs are upstream molecules of CGs, which means that CGs are regulated by ncRNAs. In addition, lncRNAs regulate miRNAs. Notably, this result is usually achieved by lncRNAs acting as molecular sponges. When CRDs occur, the human body activates ncRNAs in a uncertain way, thereby regulating the expression level of CGs. Abnormal levels of CGs expression ultimately lead to the occurrence of EC. From a macro perspective, long-term CRDs can cause EC. It is noteworthy that *NAPS2* may be regulated by *mi-93-5p* and is negatively correlated, while lncRNA *SNHG14* is also negatively correlated with *mi-93-5p*. This implies that *NAPS2* is positively correlated with lncRNA *SNHG14*. Then, according to Zhang et al. (88), lncRNA *SNHG14* is lowly expressed in EC patients, which means that *NPAS2* should also be at low expression levels. However, the study by Zheng et al. (47). demonstrated that elevated levels of *NAPS2* in EC patients. This conclusion is contradictory to their relationship. So far, we cannot explain this result, and we speculate that it may be the result of the action of multiple molecular pathways.

Interestingly, data reported by Costas et al. (89) did not support the carcinogenic role of CRDs in EC. This result is contrary to the findings of our previously mentioned study by Viswanathan et al. (69): the Nurses’ Health Study I found a significant elevated risk (RR=1.47) of endometrial cancer among long-term rotating night workers (>20 years). We observed that the study by Costas et al. (89). included only 180 cases while the study by Viswanathan et al. (69). included 53,487 women. The number of samples may have had an impact on the results of the study, and we believe that the results of a study with a large sample may be more convincing. What’s more, night work was defined as a working schedule that involved partly or entirely working between 00:00 and 06:00, while the latter defined night work as working at least three nights per month, in addition to daytime or evening shifts in that month. It can be seen that the target populations of these two studies are fundamentally different. It is possible that this is one of the reasons for the inconsistency of these two results. Although it is unclear why similar studies have reached inconsistent conclusions, the mechanism underlying the occurrence and development of EC needs to be further explored to resolve the conflicting evidence.

## 7 Conclusion and prospects

Circadian rhythms enable organisms to move regularly and maintain the balance between action and recovery, as disrupting this balance may lead to disease progression. We attempted to synthesize existing information on the role of CGs, miRNAs, and lncRNAs in developing EC to enhance our understanding of their participation in disease biology. This review summarizes the carcinogenic pathways associated with circadian gene ncRNAs. Deepening our understanding of these pathways is crucial for future EC research and can be extended to studies of other tumors. In addition, the carcinogenic pathway of CGs and ncRNAs provides a new direction for exploring new therapeutic targets. We believe that this area should be explored in more detail in the future.

## Author contributions

LZ: Data curation, Investigation, Methodology, Supervision, Writing – original draft, Writing – review & editing. SC: Supervision, Writing – review & editing. LZ: Writing – review & editing. QH: Supervision, Writing – review & editing. JC: Supervision, Writing – review & editing. WC: Supervision, Writing – review & editing. SL: Funding acquisition, Investigation, Methodology, Supervision, Writing – review & editing. QS: Funding acquisition, Methodology, Supervision, Writing – review & editing.
